# Metabolomics Discovers Early-Response Metabolic Biomarkers that Can Predict Chronic Reproductive Fitness in Individual *Daphnia magna*

**DOI:** 10.3390/metabo8030042

**Published:** 2018-07-23

**Authors:** Nadine S. Taylor, Alex Gavin, Mark R. Viant

**Affiliations:** School of Biosciences, University of Birmingham, Edgbaston, Birmingham B15 2TT, UK; nadine.taylor@cea-res.co.uk (N.S.T.); ajsgavin@gmail.com (A.G.)

**Keywords:** DIMS, direct infusion mass spectrometry, omics, toxicogenomics, OECD test guideline, AOP, key event, adverse outcome, MoA, PLS regression

## Abstract

Chemical risk assessment remains entrenched in chronic toxicity tests that set safety thresholds based on animal pathology or fitness. Chronic tests are resource expensive and lack mechanistic insight. Discovering a chemical’s mode-of-action can in principle provide predictive molecular biomarkers for a toxicity endpoint. Furthermore, since molecular perturbations precede pathology, early-response molecular biomarkers may enable shorter, more resource efficient testing that can predict chronic animal fitness. This study applied untargeted metabolomics to attempt to discover early-response metabolic biomarkers that can predict reproductive fitness of *Daphnia magna*, an internationally-recognized test species. First, we measured the reproductive toxicities of cadmium, 2,4-dinitrophenol and propranolol to individual *Daphnia* in 21-day OECD toxicity tests, then measured the metabolic profiles of these animals using mass spectrometry. Multivariate regression successfully discovered putative metabolic biomarkers that strongly predict reproductive impairment by each chemical, and for all chemicals combined. The non-chemical-specific metabolic biomarkers were then applied to metabolite data from *Daphnia* 24-h acute toxicity tests and correctly predicted that significant decreases in reproductive fitness would occur if these animals were exposed to cadmium, 2,4-dinitrophenol or propranolol for 21 days. While the applicability of these findings is limited to three chemicals, they provide proof-of-principle that early-response metabolic biomarkers of chronic animal fitness can be discovered for regulatory toxicity testing.

## 1. Introduction

Chemical safety regulations are designed to protect human and environmental health. Yet around the globe, they fail to guarantee safe products and safety from exposure to pollutants. The disease burden associated with environmental chemical exposures likely exceeds 10% of the global gross domestic product [1]. Improvements in prognostic chemical safety—that is, early testing before adverse impacts on humans and the environment occur [2,3,4]—remains a critical need of society. Yet prognostic chemical risk assessment remains entrenched in experimental procedures that fail to benefit from the genomics revolution, i.e., fail to understand the underpinning molecular mechanisms that can provide molecular diagnostic tools [5,6]. In short, there remains almost total reliance on animal testing to measure ‘apical endpoints’, i.e., safety thresholds that are based on the concentrations at which a chemical induces pathological observations in the whole organism [7].

For ecological risk assessment, chemicals are routinely tested to measure adverse impacts on acute lethality (e.g., OECD Test No. 202: *Daphnia* sp. Acute Immobilization [8]) and, of greater relevance to low dose environmental exposures, the impacts of chemicals on reproduction over chronic timescales. For example, OECD Test No. 211 (*Daphnia magna* Reproduction Test [9]) evaluates the reproductive fitness of a keystone freshwater invertebrate over 21 days, equating to approximately 5 cycles of off-spring production through one-third of this species’ life span [10]. The ecological importance of such a chronic (or long term) test is undeniably high, with reproductive fitness essential for species continuation and used as a measure in population ecology and the assessment of ecosystem health [11,12]. Yet measuring chronic effects such as reproductive toxicity by practically observing impacts on species over long timescales (i.e., 21 days for *Daphnia*; typically, 30 days for fish toxicity, OECD Test No. 210; 90 days for rodent toxicity, OECD Test No. 408 [7,9,13]) is highly resource intensive and hence expensive [14].

Furthermore, crudely counting the number of living offspring produced from *Daphnia* in OECD Test 211 conveys no knowledge of the underlying mode of action (MoA) of a chemical. Yet knowledge of MoA can have major benefits for chemical safety testing; for example, it can be used to group chemicals into similarly acting substances and more extensive animal testing can then be focused on a few representative members of that chemical group, reducing the number of animal tests (termed biological ‘read-across’ [15]. Knowledge of MoA can also serve as a source of (molecular) biomarkers for that toxicity pathway [16]. Introduced in 2010, Adverse Outcome Pathways (AOP) have provided a framework to enable a more mechanistic and causal understanding of how chemicals perturb normal biological functions, from a molecular initiating event (MIE) through to an adverse outcome (AO; i.e., apical endpoint) that affects individual organisms and, subsequently, populations [17,18,19]. One of the major challenges for AOPs is how to discover the molecular key events (KEs) that are causally predictive of these higher levels of biological organization. Indeed, this is a long-standing challenge of molecular biomarker research, to predict whole organism fitness from molecular measurements [20].

Having introduced the relevance of reproductive fitness as a measure in ecological risk assessment, yet the inadequacies of current chronic testing regimes in terms of cost and lack of mechanistic insights, we need to ask what could be a viable solution for modern chemical testing strategies. One solution would be the application of early molecular predictors of chronic reproductive toxicity, where those molecular predictors have a mechanistic basis, i.e., are biologically plausible. Practically, this would greatly reduce the need for chronic, long term animal testing, thereby reducing costs. In AOP terminology, this proposed solution is to use molecular KEs to predict individual effects such as reproductive fitness. Given this, the grand challenge is how to discover a small panel of molecular KEs for regulatory testing purposes from the tens of thousands of expressed genes, proteins and metabolites within cells and to ensure that panel of molecular KEs is predictive of the adverse outcome. Untargeted ‘omics technologies may provide one solution, which in combination with multivariate statistics are designed specifically to discover changes within the molecular landscape without bias [21]. Indeed, ‘omics approaches are increasingly being applied to a range of applications in toxicology [22,23,24], including for read-across [25] and deriving points of departure [26,27]. For the specific challenge of discovering molecular KEs that are predictive of a whole organism response—the focus of the current work—the experimental design is, however, just as critical as the measurement technology.

Practically, two experimental designs can be envisioned to discover early-response biomarkers: first, to seek to discover putative molecular KEs occurring at the same chronic time point that the adverse outcome occurs (i.e., decreased reproductive fitness), and then to evaluate whether those putative KEs can also predict decreased fitness when measured early in the exposure period. This approach benefits from a likely greater association of the molecular changes to the apical endpoint, but the successful application of the putative KEs to early exposure times rests on the assumption that these early molecular changes persist through the exposure period. The second practical strategy would be to discover the molecular changes at an early time following chemical exposure, where that exposure concentration is known to decrease reproductive fitness on a longer time scale. This approach benefits from knowing that the molecular changes do indeed occur on an early timescale, but assumes that these early molecular KEs are causally associated with the longer term apical endpoint. In the current study we have investigated the first of these two strategies. Furthermore, we have focused specifically on utilizing metabolomics to discover early metabolic KEs. This is because changes in metabolic biochemistry, as measured using untargeted metabolomics, are the most downstream of gene expression and protein translation, and represent the most functional measure of an organism’s physiology and response to toxic stress [22,23,24,28,29].

In previous work, we and others have sought to discover associations across levels of biological organization, specifically linking metabolism with whole organism apical endpoints. For example, using prior knowledge of the MoA of eight model toxicants, energy-related biomarkers (cellular energy allocation, lipid content, anaerobic metabolic activity) and parameters related to oxidative stress (measured after 48–96 h) were shown to predict population level responses in *D. magna* (after 21 days), although no attempt was made to discover the most predictive molecular KEs using an ‘omics approach [30]. Hines et al. [31] reported the first successful application of metabolomics to discover novel metabolic biomarkers that could predict the energetic fitness of marine mussels following a 7-day exposure to two model toxicants. More recently, statistical models were derived from chronic metabolic profiles that could predict reproductive impairment in adult *D. pulex-pulicaria* following exposure to both single and binary mixtures of copper and nickel, and these metabolic profiles were shown to be predictive of chronic impairment when applied to animals that had experienced only an acute exposure [32]. The metabolic profiles were not identified and the authors acknowledged that further work is required to validate this exciting finding, including evaluating this strategy to discover early-response biomarkers using more diverse chemicals and MoAs.

**Figure 1:**
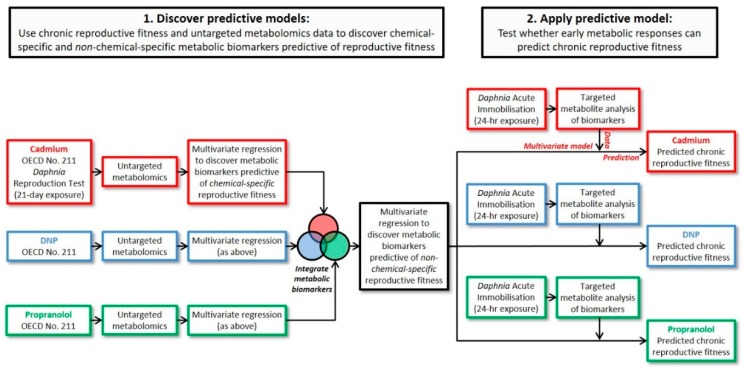
Overall workflow comprising of two *Daphnia* toxicity tests (chronic reproduction and acute immobilization), untargeted metabolomics, targeted metabolite analysis and multivariate modelling, ultimately leading to predictions of the chronic reproductive fitness of *Daphnia* from early-response metabolic biomarkers.

The overarching aim of the current study was to discover early-response metabolic biomarkers that could predict the chronic reproductive fitness of individual *D. magna*, using mass spectrometry metabolomics. To build on our earlier studies, we investigated three further model toxicants that have different MoAs: cadmium (Cd), primarily causing oxidative stress; 2,4-dinitrophenol (DNP), an uncoupler of oxidative phosphorylation; and propranolol, a non-selective beta adrenoceptor blocking drug. *Daphnia* was selected as the study species due to its importance in regulatory toxicity testing; indeed, this species is the most commonly used test organism in aquatic toxicology worldwide [33], enhancing the potential translation and impact of this research. *Daphnia* have increasingly been studied using metabolomics [24,34,35,36,37], including for elucidating chemical MoAs and building predictive models for chronic effects, both with the potential to contribute to constructing AOPs [29,32]. The current study was comprised of four specific objectives, built around a workflow summarized in Figure 1 and comprising of an OECD chronic reproductive study, an acute exposure study, untargeted metabolomics, targeted metabolite analysis and multivariate modelling. First, to ensure that all biomarker discovery was rigorously anchored to a regulatory-relevant adverse outcome, range-finding studies were conducted to determine appropriate exposure concentrations for each chemical. Second, we sought to discover putative metabolic biomarkers of reproductive fitness in *D. magna* by applying untargeted metabolomics on the final day of OECD Test No. 211 (*Daphnia* chronic reproduction) conducted for Cd, DNP and propranolol. In addition to building predictive models of reproductive fitness for each chemical individually, the third objective was to integrate the discoveries from these exposure studies of Cd, DNP and propranolol to reduce the list of putative metabolic KEs to non-chemical-specific predictors of reproductive fitness. Finally, we sought to evaluate these biomarkers, which were discovered in chronic exposure studies, for their ability to predict *Daphnia* reproductive fitness (i.e., after 21 days) from an independent metabolic dataset measured using 24-h exposures to Cd, DNP and propranolol. That is, to determine whether we had discovered early-response metabolic biomarkers that could predict the chronic reproductive fitness of individual *D. magna*.

## 2. Results

### 2.1. Chronic Chemical Exposures Reduced Daphnia Reproductive Fitness

**Figure 2:**
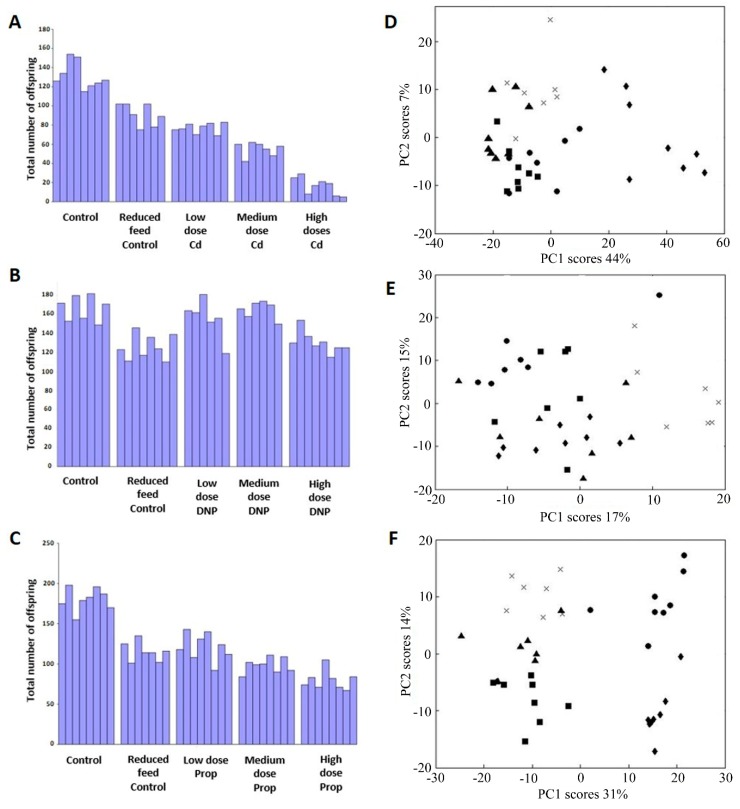
Bar charts depict the reproductive output of the same individual *D. magna* following their exposure to (**A**) Cd, (**B**) 2,4-dinitrophenol (DNP) and (**C**) propranolol. Grubbs tests determined that the reproductive output from two individuals in the DNP exposure groups were outliers (one in low dose and one in medium dose) and these have been removed from the data. PCA scores plots from analysis of mass spectrometry metabolomics data of individual *D. magna* following chronic (21-day) exposure to (**D**) Cd, (**E**) DNP and (**F**) propranolol. Classes comprise of control (▲), reduced feed control (✕), Low dose (■), medium dose (●) and high dose(s) (◆). All plots show PC1 against the (next) most significant PC axis, and all show a chemical-induced metabolic perturbation.

The first objective of the study was to ensure that all (subsequent) biomarker discovery would be rigorously anchored to a regulatory-relevant adverse outcome, specifically, a decrease in reproductive fitness (Figure 1). Initial dose range-finding studies were performed using 24-h exposures to derive the lethal exposure concentrations (neonatal LC_50_) of Cd, DNP and propranolol (see Supplementary Materials) to guide the dose selection for the 21-day *D. magna* reproduction tests (OECD Test No. 211 [9]). Using these data, chronic 21-day exposures were conducted using three concentrations of each chemical, a reduced-food group (50% feed level), and untreated controls: the chemical exposure concentrations corresponded to 0.05–10% of the neonatal LC_50_ (for each chemical), see Table S1, and all chemicals included an exposure concentration of 1% of LC_50_ for comparative purposes. Importantly, each biological replicate comprised of a single female *D. magna* in an individual exposure vessel, which had implications for the metabolomics assays described below. This design was necessary to enable the reproductive fitness of individual animals to be measured in terms of the total number of offspring they each produced during the 21-day exposure period (following the OECD test guideline). Figure 2 depicts the reproductive output for all surviving animals (each female *Daphnia* corresponds to one vertical bar) for each of the three exposure chemicals, the reduced-food, and untreated controls. Visual interpretation of the plots shows a distinct reduction in reproductive output with increasing concentrations of Cd (Figure 2A) and propranolol (Figure 2C) exposures, yet a relatively smaller effect was observed following exposure to DNP (Figure 2B). Univariate statistical analyses determined significant effects on *D. magna* reproductive output for all three chemicals: Cd (ANOVA, *p* = 1.73 × 10^−20^), DNP (*p* = 1.49 × 10^−6^) and propranolol (*p* = 1.52 × 10^−15^) with Tukey-Kramer post-hoc tests revealing significant differences in reproductive fitness as follows: Cd—all treatment groups and untreated control group were significantly different except between the reduced-food and low dose group; DNP—control, low and medium dose groups were significantly different to the reduced-food and high dose groups; propranolol—untreated control group was significantly different from all other treatment groups, with the reduced-food and low dose groups also different from the medium and high dose groups. The observations that some of the chemical exposure groups reduced reproductive fitness to a greater extent than that caused by reducing the food availability suggests a direct impact of the chemicals, i.e., the observed decreases in reproductive fitness following chemical exposure were not simply caused by reduced food intake. The metabolomics data, below, offers further insights into the reduced-food effects. Overall, the observations of significantly decreased reproductive fitness for all three chemicals provided the ‘adverse outcome’ to which the molecular biomarker discovery could be anchored.

### 2.2. Metabolomics Discovers Biomarker Signatures that Predict Daphnia Reproductive Fitness Following Chronic Exposures to Individual Chemicals

We next sought to discover putative metabolic biomarkers of reproductive fitness in *D. magna* by applying untargeted metabolomics to the same individual animals used in the reproduction tests (from above; Figure 1). It was therefore essential that our metabolomics assays were sufficiently sensitive to measure samples of ca. 1.5 mg biomass, so that associations could be discovered between metabolic signatures and reproductive fitness in the same individuals. We selected nanoelectrospray direct infusion mass spectrometry (DIMS) as it provided the necessary analytical sensitivity [38], yielding mass spectra comprising of >3500 peaks (after removal of noise and peaks from the extract blank).

Univariate and unsupervised multivariate analyses of the metabolomics data were applied to provide an initial assessment of any metabolic perturbations induced by each of the three chemicals. FDR-corrected univariate analyses reveal that significant metabolic perturbations were caused by chemical exposure (Table S2). Visual interpretation of principal components analysis (PCA) scores plot for each chemical shows a clear separation between the treatment groups following exposure to Cd and propranolol (Figure 2D,F, respectively), consistent with the univariate analyses (Table S2). Exposure to DNP results in less well separated groups using PCA (Figure 2E), consistent with the univariate analyses (Table S2) and the modest reduction of reproductive fitness (Figure 2B). For all three chemicals, the reduced-food group was significantly separated from the chemical-exposed groups confirming that the chemicals were not inducing a starvation phenotype in the *Daphnia* (see Supplementary Materials; Table S3). These molecular insights allowed us to conclude that the observed decreases in reproductive fitness following chemical exposure (Figure 2A–C) were not simply caused by reduced food intake. The reduced-food group could therefore be removed from the subsequent biomarker discovery analyses.

**Figure 3:**
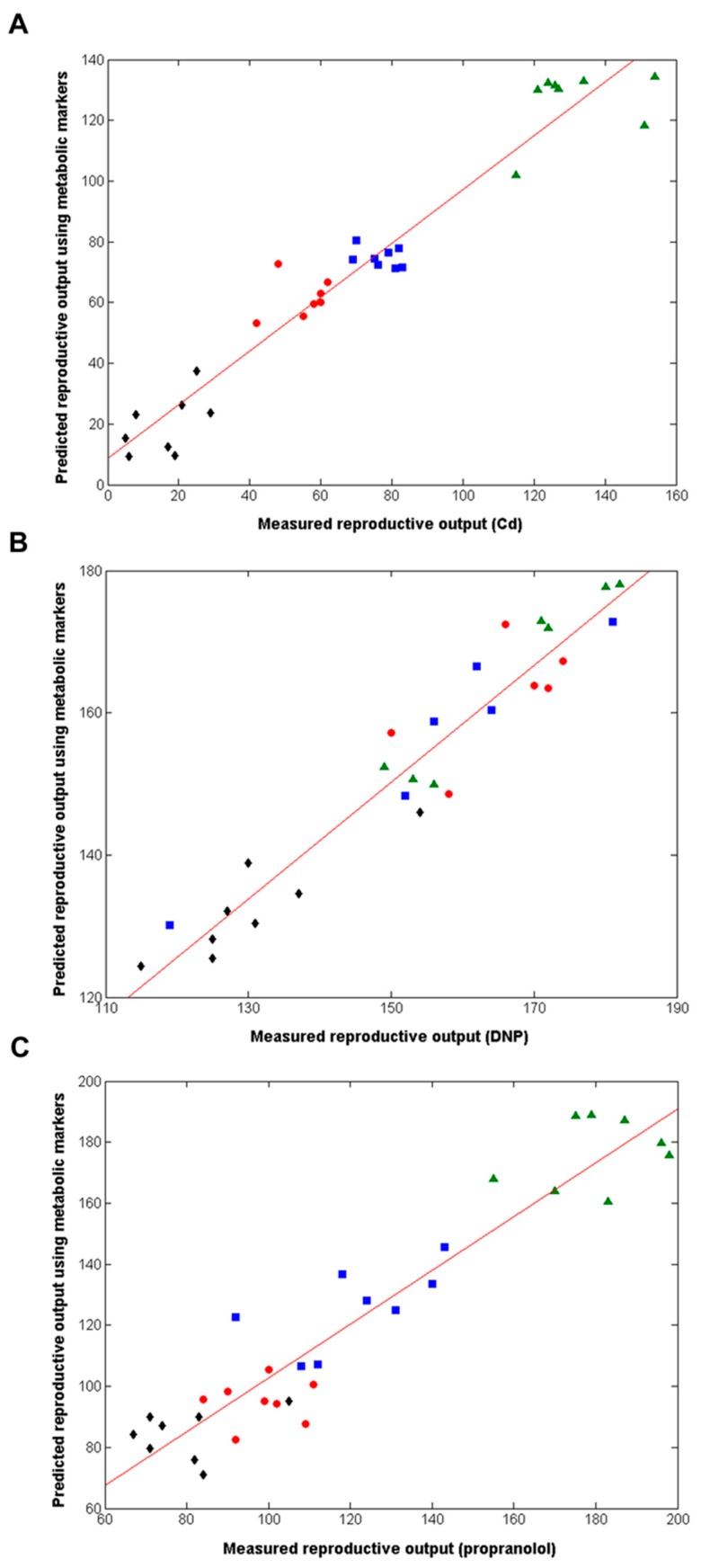
Correlation between measured and predicted reproductive output for individual *D. magna*, the latter derived from forward-selected PLS-R models built using the metabolomics datasets, following chronic exposure to (**A**) Cd, (**B**) DNP and (**C**) propranolol, including lines of best fit. The cross-validated *R*^2^ values are 0.935 for Cd (561 peaks, 2 LVs), 0.945 for DNP (306 peaks, 3LVs) and 0.893 for propranolol (606 peaks, 3LVs); *p* = < 0.001 in all cases. Classes comprise of control (▲), Low dose (■), medium dose (●) and high dose(s) (◆).

Next, we employed supervised multivariate analyses to attempt to discover metabolic signatures that could predict the reproductive fitness of *D. magna* (Figure 1). Partial least squares regression (PLS-R) was applied to each of the three metabolomics datasets (Cd, DNP and propranolol), and the models built were internally cross-validated (i.e., evaluated as to how well they predict reproductive output for samples left out of the original model building). Strong associations between the measured and predicted reproductive outputs were obtained: cross-validated *R*^2^ value of 0.822 for Cd (4056 peaks in PLS-R model); *R*^2^ = 0.768 for DNP (4112 peaks); *R*^2^ = 0.735 for propranolol (3647 peaks). Permutation testing was applied to assess the statistical significance of the capability of each PLS-R model to predict the reproductive output; for all chemicals, *p* < 0.001, i.e., none of the 1000 permuted PLS models generated *R*^2^ values that were higher than those from the models built with the real metabolomics data. While these initial PLS-R models were robust, they generated long lists of putative metabolic biomarkers. We sought a reduced list of the most predictive metabolites to translate towards practical regulatory applications. Therefore, next we employed a ‘forward selection’ approach in our PLS-R model building to derive the most reduced list of putative metabolic biomarkers that built an optimally predictive model for each chemical, i.e., the model with the highest *R*^2^ value. The optimal individual-chemical PLS-R models are shown in Figure 3, with cross-validated *R*^2^ = 0.935 for Cd (561 peaks in model); *R*^2^ = 0.945 for DNP (306 peaks); *R*^2^ = 0.893 for propranolol (606 peaks); and *p* < 0.001 for all models (permutation testing). These results provide very strong evidence for the existence of metabolic predictors of reproductive fitness, i.e., metabolic predictors of the number of offspring from individual female *Daphnia* produced in a 21-day OECD Test No. 211, for each of Cd, DNP and propranolol.

### 2.3. Metabolomics Discovers a 49-Biomarker Signature that Predicts Reproductive Fitness Following Chronic Exposure to All Three Chemicals

**Figure 4:**
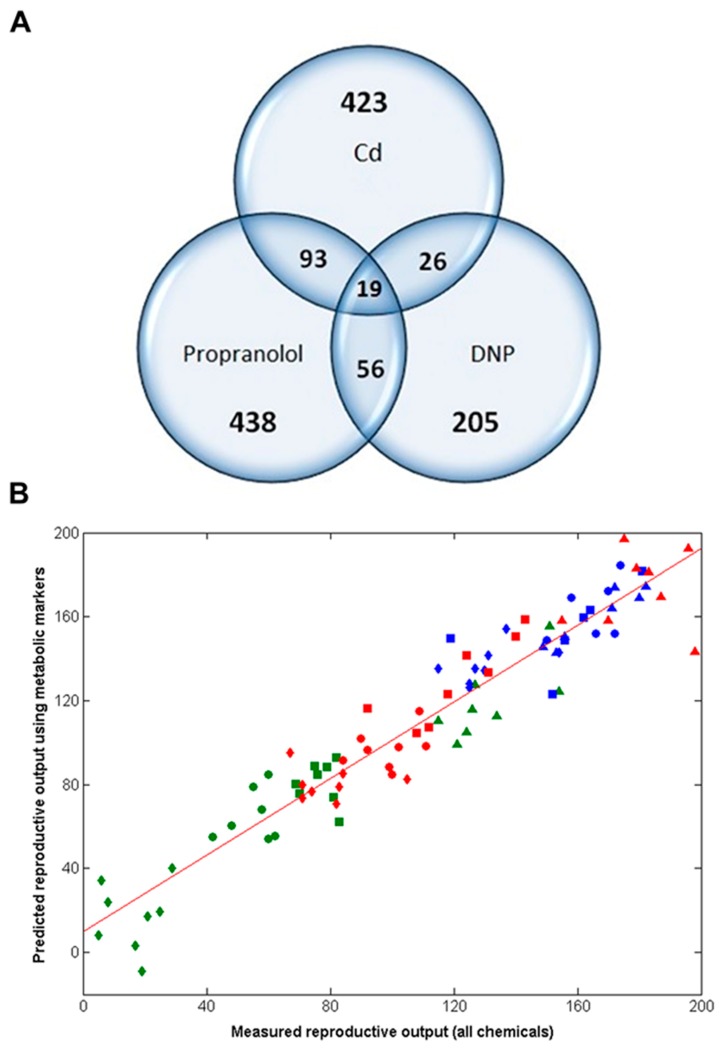
(**A**) Venn diagram depicting the relationship between the metabolic features (i.e., peaks) from each of the optimal PLS-R models following chronic exposure of Daphnia to Cd, DNP or propranolol; (**B**) Correlation between measured and predicted reproductive output for individual D. magna, the latter derived from the non-chemical-specific forward selected PLS-R model, following chronic exposure to Cd, DNP and propranolol, including line of best fit. The cross-validated *R*^2^ value is 0.915 (49 peaks, 3LVs); *p* = < 0.001. Classes comprise of Cd exposed animals: control (▲), low (■), medium (●) and high dose (◆); DNP exposed: control (▲), low (■), medium (●) and high dose (◆); propranolol exposed: control (▲), low (■), medium (●) and high dose (◆).

The analyses above discovered three highly predictive models of *Daphnia* reproductive fitness. Yet the domains of applicability of each of these three models should be viewed as limited to each of the three exposure chemicals, Cd, DNP and propranolol; i.e., we cannot assume that the model built with Cd is valid for all chemicals inducing oxidative stress, etc. However, the domain of applicability of this study can be increased significantly by determining if there are any putative metabolic biomarkers that are common to all three chemicals. If these exist, they can be viewed as a non-chemical-specific (or non-MoA-specific) biomarker signature of reproductive fitness in *Daphnia*. Therefore, the metabolic features from the three optimal individual-chemical PLS-R models were compared (Figure 1 and Figure 4A), revealing that 194 metabolic features were common to at least two of the three chemicals (using an *m*/*z* tolerance of 1.5 ppm to combine the lists). Next, to attempt to reduce this putative metabolic biomarker list still further, one further PLS-R model was built using forward selection applied to just these 194 metabolic features. The optimal, non-chemical-specific PLS-R model is shown in Figure 4B, with a cross-validated *R*^2^ of 0.915 and only 49 peaks in the model. Considering that >4000 peaks were detected using the DIMS metabolomics approach, the strategies applied here have focused on the ca. 1% most important metabolic features only. Table S4 lists the 49 peaks in ranked order of importance in the PLS-R model as well as their *m*/*z* values, average intensities and the fold-change in intensities between the high dose and control groups (for each of the three chemicals).

Biological plausibility is one of the criteria for robust molecular KEs within AOPs, which means that molecular biomarkers should be identified. As is widely recognized in metabolomics, metabolite identification can be extremely challenging. For example, to confidently identify a metabolite to the Metabolomics Standards Initiative (MSI) level 1, the experimental measurements of the metabolite in the biological sample need to be validated against the same type of measurements using an authentic chemical standard [39]. Yet for most metabolites, such standards are not commercially available. *De novo* synthesis of a single or a few metabolites is an option for specialist laboratories [40], but this does not scale even to a few tens of metabolites. In the current study, we putatively annotated as many as possible of the 49 metabolic features to at least MSI level 2, as described in Materials and Methods. Table S5 summarizes these annotations including the measured *m*/*z* values, selected correlations (only those *r* > 0.9) between the intensities of pairs of peaks, the (putative) empirical formula(e), ion form (i.e., in which adduct form and isotope the metabolite was measured), *m*/*z* error compared to theoretical mass (ppm), the observation of related adduct forms and naturally-occurring isotopes to support the peak annotations, and where known the putative metabolite name. Metabolite identification to MSI level 1 was conducted by comparing MS/MS fragmentation spectra to those measured using a commercial authentic metabolite standard. Table 1 summarizes the annotations and identifications for the sub-group of metabolic features that could be assigned a unique empirical formula and, where possible, (putative) metabolite name, which accounted for 11 of the 49 peaks in the biomarker signature.

### 2.4. Chronic Reproductive Fitness Can Be Predicted from Early-Response Metabolic Measurements

**Figure 5:**
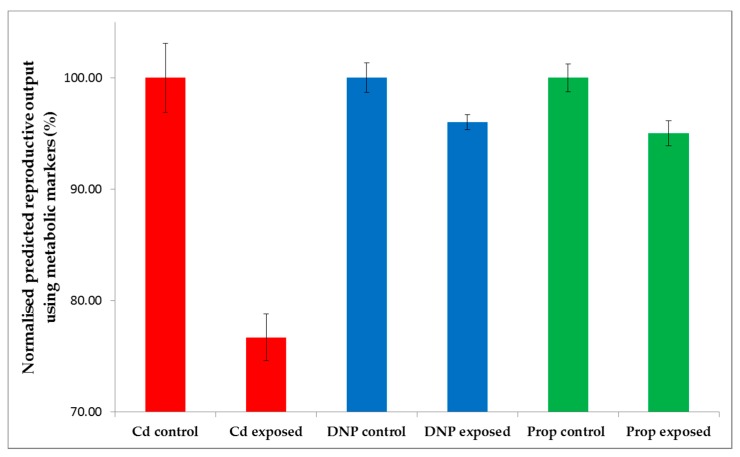
Predictions of the reproductive outputs for individual *D. magna* following hypothetical 21-day exposures to Cd, DNP and propranolol, relative to untreated controls, derived by submitting three previously published acute toxicity metabolomics datasets [29] to the non-chemical-specific forward selected PLS-R model from this study. Reproductive outputs are normalized for each chemical, with the control group set at 100%. Error bars represent ±SEM.

Considering this study within the context of the international 21-day *Daphnia* reproductive toxicity test, the capability of the 49-biomarker signature to predict the number of offspring using metabolite data measured on day 21 is at best interesting, but of no practical value to chemical regulators. As introduced above, however, this biomarker signature would have considerably greater value if it could predict chronic reproductive toxicity based upon metabolic measurements derived from a (much lower cost) short term study. If successful, this would greatly reduce the need for chronic, long term animal testing, thereby reducing costs. We therefore applied the newly discovered 49-biomarker signature to our previously published metabolomics datasets for *D. magna*, corresponding to early-response (24-h) exposure studies of Cd, DNP and propranolol [29] (Figure 1). These 24-h exposures were conducted at a single dose of 10% LC_50_, which we know would cause a statistically significant decrease in reproductive output if the studies had been run for 21 days (Figure 2B-C for DNP and propranolol). Note that for the case of Cd in Figure 2A, reproductive output was measured for exposure concentrations up to 1% LC_50_ (Table S1), which caused a significant decrease; here we assume that an even higher exposure concentration of 10% LC_50_ for Cd would also result in a significant decrease in reproductive output. In fact, this is critical knowledge for Cd, DNP and propranolol, which we rely upon for determining whether the predictions of chronic adverse outcome from the early-response metabolic measurements are correct or not. Of the 49 metabolic features in the biomarker signature discovered in this study, 39 of these were observed in the early-response Cd data, 36 in the early-response DNP and 35 in the early-response propranolol datasets. The intensities of any missing peaks in these datasets were set to zero. Next, each of the three early-response toxicity datasets were submitted to the optimal, non-chemical-specific PLS-R model, which predicted the number of *Daphnia* offspring produced following hypothetical 21-day exposures. Figure 5 summarizes the predicted reproductive output (with control animals normalized to 100%) for Cd, DNP and propranolol. Univariate statistical analyses of these predictions (see section 4.5) confirmed that they correctly predict a decrease in reproductive fitness: Cd (*t*-test, *p* = 6.93 × 10^−6^), DNP (*p* = 0.0154), propranolol (*p* = 8.12 × 10^−3^).

## 3. Discussion

Given the importance of *Daphnia* as a test organism in regulatory toxicology [41], and as a model organism in freshwater ecology [42] and more recently human health research through its adoption as a US National Institutes of Health model organism [43], the relative lack of knowledge of the identities of the polar metabolites and lipids in this species is arguably surprising. Researchers from different fields may use different terms: toxicologists may ask what is known of *Daphnia’s* metabolic biochemistry; ecologists may enquire about its chemical ecology; while “omics” investigators may ask what is known of *Daphnia’s* metabolome? Yet these questions all refer to the same natural compounds, i.e., the primary and secondary metabolites that are ingested, synthesized and/or excreted by *Daphnia*. To date, only a few hundred metabolites in *Daphnia* have been reported in the literature, for example see references [37,44]. The challenges of annotating and/or identifying the peaks measured in a metabolomics study are clear from the current work. While an array of analytical measurements (accurate *m*/*z* with <1 ppm accuracy [45], MS/MS) and data analysis approaches (interpretation of adduct patterns, isotope patterns, correlation of peak intensities [46], etc.,) have been applied, only 11 of the 49 peaks in the biomarker signature of reproductive fitness have been identified. Clearly more sophisticated approaches are required to identify metabolomes. One strategy that is currently being developed is ‘Deep Metabolome Annotation’, an integrated analytical, computational and informatics workflow that seeks to extract, characterize and store knowledge of both polar and lipophilic metabolites from a cell, tissue or whole organism. This approach combines solid phase extraction, non-targeted LC-MS(/MS), GC-MS, NMR and nanoelectrospray ionization DIMS^n^ with an array of data analysis algorithms and informatics resources and is currently being used to extend the annotations and identification of the *D. magna* metabolome [47].

We argue that applying such large-scale annotation and identification is critical for regulatory toxicology, as meaningful biological inferences may only be drawn from metabolomics datasets where peaks can be definitively named. Practically, to translate discoveries from a metabolomics study in toxicology into a practical metabolic KE that could be deployed in a targeted high throughput toxicity screening assay is going to require identification of that or those metabolites. One further strategy to address this challenge was recognized in 2015 by the formation of a scientific task group of the international Metabolomics Society to progress the characterization of metabolomes, specifically focusing on a small number of model organisms [48]. The value of model organisms across biology and medicine is very well established [49]. Motivated by the challenges we encountered in the current discovery study, we strongly advocate for the deep metabolome annotation and identification of the most widely used test species in (regulatory) toxicology including in vitro models.

The annotation and identification of the 49-biomarker metabolic signature, albeit limited in completeness, has in fact produced some intriguing findings. Ascorbate (ascorbic acid or more commonly, vitamin C) is one of the major water-soluble antioxidants present in animals and plants [50,51]. Ascorbate decreased in concentration in response to all chemical treatments (average fold change from control to high dose: 0.498 (Cd), 0.636 (DNP) and 0.571 (propranolol); Tables S4 and S5), most likely indicating the *Daphnia* experienced oxidative stress. Glutathione (GSH), the second common water-soluble antioxidant, is not a component of the final 49-biomarker signature but was also detected in our metabolomics study. Table S6 shows the effects of chemical exposure on *Daphnia* GSH levels and confirms that oxidative stress was occurring following Cd exposure, as would be expected based on its primary MoA (average fold change from control to high dose Cd of 0.652). GSH levels were increased following DNP exposure (1.55-fold) and unchanged by propranolol (0.969-fold; Table S6). The differing response of GSH across the three chemicals is the most likely cause for its exclusion from the final biomarker signature by the PLS-R feature selection algorithm.

While this perturbation of antioxidants was expected [52], given the inclusion of a heavy metal in the study, the observation of four sulfonated lipids in the list of putatively annotated metabolites was not (Table 1). Although the structures of the C_c_H_h_SO_4_, C_c_H_h_SO_5_ and C_c_H_h_SO_6_ metabolites could not be determined unambiguously, the MS/MS fragmentation studies revealed that these molecules contain lipophilic hydrocarbon backbones and a sulfate group. The functions of such compounds can be hypothesized from the literature. Amphipathic structures (i.e., polar and non-polar regions within the same molecule) are consistent with these molecules acting as anionic surfactants, and similar endogenous surfactants have been reported previously within the guts of aquatic invertebrates to induce micelle formation and enhance the solubilization of food [53,54,55]. The digestive juice of the land snail *H. pomatia L.* has also been reported to contain alkyl sulfates [56]. This suggests that part of the 49-biomarker signature of reproductive fitness could be linked to early changes in gut digestive physiology in *Daphnia*. While speculative, there is a logical basis for associations between digestion and reproduction, namely the allocation of energy resources. Chemical exposure can affect the metabolic processes involved in energy production, storage and metabolism, processes which are fundamental to organism growth and reproduction. It has been shown in *Daphnia* that a toxic assault can lead to changes in energy allocation that is highly correlated with impaired reproduction [30,57]. Therefore, further investigations of this potentially important class of sulfonated lipids and their ability to predict reproductive dysfunction in *Daphnia* is warranted.

For the strategy of metabolomics biomarker discovery presented in the current study to be translated successfully to regulatory toxicology, not only do we need to improve our ability to identify metabolites, but we also require biological validation of any putative biomarker signatures that are discovered. Obviously, the metabolomics measurements and statistical analyses need to be conducted rigorously, including for example the use of cross-validation and permutation testing (both used here) when building predictive multivariate models. In addition, more rigorous biological validation would require that this whole study is repeated independently, and the new biomarker signature checked for consistency with the one reported here. This level of biological validation was not conducted in the current study. Following that additional study, for putative biomarkers to advance towards becoming established metabolic KEs they should be validated using independent toxicity studies. The strategy that we would recommend is to develop targeted assays to measure just the metabolic biomarkers of interest, using LC-MS/MS. If the physico-chemical properties of the biomarkers within the signature are quite different, this might require two or more LC separation methods. Targeted LC-MS/MS measurements of the selected biomarker signature would first require analytical validation of the method (e.g., linearity, accuracy, precision, limits of detection and quantification, etc.) as well as biological validation by measuring the biomarkers in multiple new (i.e., independent) samples. What is noteworthy from the current study is the number of metabolic features in the biomarker signature. The most predictive biomarker signature contained 49 metabolic features, i.e., it was not a signature with only one or a few metabolites (arguably a failing of earlier biomarker studies to rely on single or few endpoints), nor did the discovered biomarker signature contain many hundreds of metabolites. From an analytical perspective this is highly encouraging as targeted LC-MS/MS measurements of a few 10′s of metabolites is achievable with existing mass spectrometry platforms, meaning that translation of putative biomarker signatures is not dependent on future technology solutions.

In the current study we sought to extend the domain of applicability of the biomarker signature beyond individual chemicals by integrating the discoveries from three separate chemicals and then building a multivariate predictive model to determine the subset of metabolic features that best predicted *Daphnia* reproductive output. Again, considering strategies for how the current study could be translated to regulatory toxicology, it would be critical to evaluate a much broader toxicological space, i.e., to discover putative metabolic KEs for the majority of “toxicity pathways”, and related to this to explore a much broader chemical space. To power such a study correctly, and to cover several toxicity pathways, would require at least a 10-fold increase in the scale of the experimentation, arguably several hundred chemicals would be required to cover the MoA space. From a practical perspective, this is technologically feasible, although a very high attention to quality assurance and control as well as method performance criteria would be critical to extract knowledge from a protracted metabolomics investigation. A tripartite group of scientists, spanning academic, government and industry laboratories, are now defining best practice, performance standards and reporting standards for the applications of metabolomics in regulatory toxicology [58]. This group aims to provide regulators and other stakeholders with the first practical guidelines for reporting and interpreting the quality of metabolomics data in toxicology, including for large scale investigations as would be needed to expand on the work presented here.

Yet such a significant expansion of the study described here leads directly to a further logistical need: who would be willing to pay for this? Currently, within Europe, the onus is on industry to pay for the risk assessments of the chemicals that they synthesize and/or use, so is there a business case for industry to utilize metabolomics (and other ‘omics modalities) in regulatory toxicology to a much greater degree than at present? In the context of the study presented here, replacing a standard OECD chronic *Daphnia* reproduction test with an acute *Daphnia* toxicity test design (compatible with deploying the new early-response metabolic predictors of chronic reproductive fitness) would amount to a 7- to 10-fold cost saving for industry [59]. Could this provide sufficient motivation to the chemical industry? The company BASF SE have already strongly endorsed the application of metabolomics within their chemical risk assessment paradigm, including as a tool for biological read-across [15]. Similar to the approaches presented here, the insights into the MoAs of chemicals provided by metabolomics have allowed a chronic 90-day study in rats to be reduced to only 28-days [25], with significant resource savings. In summary, we and others believe strongly in the capability of metabolomics to discover early-response biomarker signatures that are predictive of chronic adverse outcomes (i.e., apical endpoints) because metabolic measurements are the most downstream of all molecular changes and closely represent an organism’s phenotype. The discovery of a panel of metabolic markers that can predict the 21-day reproductive fitness of *Daphnia* exposed to Cd, DNP and propranolol from metabolic measurements made after only 24-h exposures adds further weight to that argument.

## 4. Materials and Methods

### 4.1. Daphnia Magna Culturing and Chemical Exposures

Cultures of *D. magna* were maintained as previously reported [38]. Individual *D. magna* neonates (3rd brood, <24-h old) in 250 mL media were exposed for 21 days to a range of concentrations of each chemical tested (*n* = 8 exposure vessels per concentration). Animals were fed daily with *Chlorella vulgaris* (no supplements) as per normal culturing conditions, with media and chemical renewal every 48 h. Three chronic toxicity studies were conducted using Cd, DNP or propranolol as the test chemicals (Sigma Aldrich, UK) prepared as described previously [29]. Nominal concentrations were: 0, 0.35, 1.4, 3.5 and 7 μgL^−1^ cadmium (measured as Cd^2+^ ions); 0, 0.15, 0.75 and 1.5 mgL^−1^ DNP; and 0, 0.14, 0.7 and 1.4 mgL^−1^ propranolol. As a means of normalizing to biological effect, the exposures of 7 μgL^−1^ cadmium, 0.15 mgL^−1^ DNP and 0.14 mgL^−1^ propranolol all corresponded to 1% of the previously determined neonatal LC_50_ (see Supplementary Materials). For each of the three chronic studies, a further control group was set up with *D. magna* fed only a half ration of algae (termed “reduced-food control”), in order to determine if observed metabolic changes were a direct effect of chemical exposure or instead a chemical-induced reduction in feeding. Throughout each of the 21-day studies, the reproductive output of individual *D. magna* was monitored and recorded, specifically the number of offspring produced per adult per brood and the day on which each brood was released. Neonates were removed from the test vessels and discarded daily; any mortality during the exposure period was also recorded. At the end of the 21-day test period, the now adult daphniids were captured and flash frozen as previously detailed [29], and then stored at −80 °C until metabolite extraction.

### 4.2. Metabolite Extraction and Mass Spectrometry Metabolomics

Metabolites were extracted from individual *D. magna* using a well-established bead-based homogenization and methanol/water/chloroform extraction protocol [29,38], including the preparation of an extract blank (same analytical procedure but no biological material). For this study, only the polar extracts were analyzed using Fourier transform ion cyclotron resonance mass spectrometry. Specifically, each daphniid extract was analyzed in triplicate on a hybrid 7-T LTQ FT mass spectrometer (Thermo Scientific, Bremen, Germany) with a chip-based direct infusion nanoelectrospray (nESI) ionization source (Triversa, Advion Biosciences, Ithaca, NY, USA), utilizing the SIM-stitching approach in negative ion mode. All data acquisition parameters were as previously detailed [38]. For each of the three metabolomics datasets corresponding to the three chemicals studied, mass spectra were processed using a three-stage peak picking and noise filter, missing value imputation, normalization and g-log transformation, as previously described [29] with some modifications. In this study the sample filter was set to retain in the final datasets only those peaks that occurred in >50% of all samples using an *m*/*z* tolerance of 1.8 ppm, and peaks in the extract blank were retained only if they were at least three times more intense in the biological samples. This workflow yielded three separate processed data matrices, one each for Cd, DNP and propranolol. Putative peak annotation and identification utilized a range of approaches, including analytical measurements (accurate *m*/*z* with <1 ppm accuracy [45] and collision-induced dissociation MS/MS) and informatics approaches. Specifically, we used custom written MI-Pack software version 1 [60] as previously detailed [29], which in addition to providing information on the *Daphnia* endogenous metabolome also enabled us to identify (and subsequently remove prior to statistical analysis) peaks directly arising from the test chemicals. Further methods to annotate and identify peaks included the interpretation of adduct patterns, isotope patterns, and highly correlated pairs of peak intensities that indicated the peaks likely arose from the same metabolite [46]). Where possible, metabolite identification to MSI level 1 [39] was conducted by comparing MS/MS fragmentation spectra to those measured using a commercial authentic metabolite standard.

### 4.3. Statistical Analysis of Reproductive Output

The total number of offspring produced per individual *D. magna* over the duration of each 21-day study was used as a measure of reproductive output. For each chemical, Grubbs statistical tests were employed for each dose group to identify any potential outliers, which were subsequently removed from any further analysis. ANOVA followed by a Tukey-Kramer post-hoc testing was then applied across all control and dose groups, per chemical.

### 4.4. Statistical Analysis of Metabolomics Datasets from 21-Day Exposures

To visualize the effects of each chemical on the metabolome of *D. magna*, principal components analyses (PCA) were conducted on the three processed and normalized (including g-log) data matrices (one per chemical). In addition, *t*-tests adjusted for a false discovery rate (FDR) <5% were applied across all peaks (metabolic features) between the untreated control and high dose group to provide an initial indication of the relative sizes of the metabolic perturbations induced by each chemical; this utilized the three processed and normalized (excluding g-log) data matrices. The most important algorithm for this study was partial least squares regression (PLS-R), a supervised multivariate method for relating two data matrices, in our case metabolomics peak intensities and *Daphnia* reproductive fitness. PLS-R derives its usefulness from its ability to analyze data with many noisy, collinear and even incomplete variables in both data matrices [61]. We utilized PLS-R modelling to discover metabolic predictors of reproductive output, for each of the three chemicals separately and also after merging the three datasets. All PLS-R models were internally cross validated, using Venetian blinds with 6 splits, to assess for any over-fitting of the data based on cross-validated *R*^2^ values [61]. In addition, permutation testing was applied, using 1000 random permutations per model built, to determine the significance of the capability of each PLS-R model to predict the reproductive output [62]. A forward selection strategy was applied to PLS-R model building to derive the minimum number of metabolic features that best predict the reproductive fitness.

### 4.5. Statistical Analysis of Metabolomics Datasets from 24-h Exposures

Metabolomics data recorded using the same DIMS metabolomics workflow, but for *Daphnia* exposed to Cd, DNP and propranolol (each *n* = 10 biological replicates) for only 24 h, were published by us previously [29] (see further Materials and Methods in Supplementary Materials) and re-examined in this study. Here we treated these early-response metabolomics datasets as ‘targeted’ metabolic measurements, i.e., we only extracted and used the intensities of the 49 peaks corresponding to the newly discovered biomarker signature (described in Results), for each chemical. This required some alignment of the *m*/*z* values across the datasets and applying a 3 ppm *m*/*z* tolerance; the intensities of any of the 49 peaks that were missing in the early-response datasets were set to zero. Then each of these ‘targeted’ metabolic measurements, one for each chemical, were submitted to the optimal, non-chemical-specific PLS-R model to predict the reproductive output of *Daphnia* following hypothetical 21-day exposures (described in Results). Predicted reproductive outputs for untreated controls and chemical-exposed groups were normalized and compared using Student’s *t*-tests on a chemical-by-chemical basis. Specifically, using Cd as the example, the predicted reproductive outputs from the PLS-R model for the Cd-treated and Cd-control groups were normalized by scaling the mean of the Cd-control group to 100%. Next a Student’s *t*-test was applied to evaluate the statistical significance of the difference in the means of the predicted reproductive outputs of the Cd-treated versus Cd-control groups.

## 5. Conclusions

The overarching aim of this study was to discover early-response metabolic biomarkers that could predict the chronic reproductive fitness of individual D. magna, which has been achieved successfully. Using the newly discovered non-chemical-specific biomarker signature of 49 metabolic features in conjunction with metabolic measurements of *Daphnia* exposed for only 24-h, we were able to correctly predict that a significant decrease in reproductive fitness of *Daphnia* would occur after 21 days. This prediction was correct for all three of the chemicals investigated, cadmium, 2,4-dinitrophenol and propranolol. Further work is needed to extend the applicability of this metabolomics discovery approach to many more chemicals and chemical classes, though our findings provide encouragement that early-response metabolic biomarkers of established apical endpoints can be discovered and translated towards regulatory toxicology.

## Figures and Tables

**Table 1:** Metabolic features within the non-chemical-specific biomarker signature that could be putatively annotated or identified with a unique empirical formula and/or metabolite name (i.e., to MSI level 1 or 2).

Rank Order of Peak Importance in PLS-R Model	Unique Empirical Formulae	Metabolite Name(s)	MSI Level	Measured *m/z* (Intensity Correlation between Peak-Pairs)	Ion Form(s)	*m/z* Error (ppm)	Supporting Peak Annotations (Other Adducts or Isotopes)
5	C_6_H_8_O_6_	Ascorbate	1 (compared to authentic chemical standard)	175.02480	[M − H]^−^	−0.08	[M + Na − 2H]^−^ *m/z* 197.00675 (−0.04 ppm error), [M + Cl]^−^ *m/z* 211.00136 (−0.62 ppm)
13, 6	C_12_H_18_O_6_	Unknown	2 (accurate *m/z*, peak correlation, isotope pattern)	257.10306, 258.10642 (*r* = 0.996)	[M − H]^−^, [M(^13^C) − H]^−^	−0.01, 0.01	−
9	C_12_H_24_SO_4_	Sulfonated lipid	2 (accurate *m/z*, MS/MS; but no standard available)	263.13227	[M − H]^−^	0.05	−
17	C_12_H_24_SO_6_	Sulfonated lipid	2 (accurate *m/z*, MS/MS; but no standard available)	295.12191	[M − H]^−^	−0.60	[M + Na − 2H]^−^ *m/z* 317.10402 (−0.03 ppm), [M + K − 2H]^−^ *m/z* 333.07797 (0.00 ppm)
23, 31	C_7_H_10_O_7_	3-Hydroxybutane-1,2,3-tricarboxylate, 2-hydroxybutane-1,2,4-tricarboxylate, 2-methylcitrate and/or homoisocitrate	2 (accurate *m/z*, peak correlation, isotope pattern)	242.01539, 243.00911 (*r* = 0.970)	[M(^13^C) + Cl]^−^, [M + (^37^Cl)]^−^	−0.09, 0.01	−
25	C_11_H_24_SO_4_	Sulfonated lipid	2 (accurate *m/z*, MS/MS, isotope pattern; but no standard available)	252.13562	[M(^13^C) − H]^−^	0.04	[M − H]^−^ *m/z* 251.13225 (−0.02 ppm)
26	C_10_H_20_SO_5_	Sulfonated lipid	2 (accurate *m/z*, MS/MS; but no standard available)	251.09579	[M − H]^−^	−0.32	−
43	C_8_H_15_NO_6_	N-Acetyl-D-galactosamine, N-acetyl-D-glucosamine and/or N-acetyl-D-mannosamine	2 (accurate *m/z*, isotope pattern)	258.05642	[M + (^37^Cl)]^−^	0.11	[M − H]^−^ *m/z* 220.08268 (0.08 ppm), [M + Cl]^−^ *m/z* 256.05935 (0.04 ppm), [M + K − 2H]^−^ *m/z* 258.03844 (−0.41 ppm)
49	C_14_H_23_N_10_OP	Unknown	2 (accurate *m/z*, isotope pattern)	415.14588	[M + (^37^Cl)]^−^	0.09	[M − H]^−^ *m/z* 377.17266 (1.44 ppm), [M + Cl]^−^ *m/z* 413.14865 (−0.35 ppm), [M(^13^C)+ Cl]^−^ *m/z* 414.15214 (−0.02 ppm), [M(^13^C)+(^37^Cl)]^−^ *m/z* 416.14882 (−0.91 ppm)

## Supplementary Materials

The following are available online at http://www.mdpi.com/2218-1989/8/3/42/s1. Supplementary methods and results for Article: Metabolomics discovers early-response metabolic biomarkers that can predict chronic reproductive fitness in individual *Daphnia magna* (includes Tables S1–S4 and S6), Table S5: Summary of all annotations for the 49 metabolic features (or peaks) that constitute the non-chemical-specific metabolic biomarker signature.

## References

[1] Grandjean P., Ballanger M. (2017). Calculation of the disease burden associated with environmental chemical exposures: Application of toxicological information in health economic estimation. Environ. Health.

[2] Darnerud P.O. (2003). Toxic effects of brominated flame retardants in man and wildlife. Environ. Int..

[3] Fry M., Toone C.K. (1981). DDT-Induced feminization of gull embryos. Science.

[4] Whitehorn P.R., O’Connor S., Wackers F.L., Goulson D. (2012). Neonicotinoid pesticide reduces bumble bee colony growth and queen production. Science.

[5] Weaver J.M., Ross-Innes C.S., Fitzgerald R.C. (2014). The ‘-omics’ revolution and oesophageal adenocarcinoma. Nat. Rev. Gastroenterol. Hepatol..

[6] Erickson A.R., Cantarel B.L., Lamendella R., Darzi Y., Mongodin E.F., Pan C., Shah M., Halfvarson J., Tysk C., Henrissat B. (2012). Integrated metagenomics/metaproteomics reveals human host-microbiota signatures of Crohn’s disease. PLoS ONE.

[7] Organization for Economic Cooperation and Development (OECD) (1998). OECD Guidelines for Testing of Chemicals, Section 4, Test No. 408: Repeated Dose 90-Day Oral Toxicity Study in Rodents.

[8] Organization for Economic Cooperation and Development (OECD) (2004). OECD Guidelines for Testing of Chemicals, Section 2, Test No. 202: Daphnia sp. Acute Immobilisation Test.

[9] Organization for Economic Cooperation and Development (OECD) (2012). OECD Guidelines for Testing of Chemicals, Section 2, Test No. 211: Daphnia Magna Reproduction Test.

[10] Pennak R.W. (1978). Fresh-Water Invertebrates of the United States.

[11] Rockwood L.L., Witt J.W. (2015). Introduction to Population Ecology.

[12] Brown J.H., Gillooly J.F., Allen A.P., Savage V.M., West G.B. (2004). Toward a metabolic theory of ecology. Ecology.

[13] Organization for Economic Cooperation and Development (OECD) (2013). OECD Guidelines for Testing of Chemicals, Section 2, Test No. 210: Fish, Early-life Stage Toxicity Test.

[14] Ankley G.T. (2008). Genomics in Regulatory Ecotoxicology: Applications and Challenges.

[15] Van Ravenzwaay B., Herold M., Kamp H., Kapp M.D., Fabian E., Looser R., Krennrich G., Mellert W., Prokoudine A., Strauss V. (2012). Metabolomics: A tool for early detection of toxicological effects and an opportunity for biology based grouping of chemicals—From QSAR to QBAR. Mutat. Res..

[16] Ankley G.T., Jensen K.M., Makynen E.A., Kahl M.D., Korte J.J., Hornung M.W., Henry T.R., Denny J.S., Leino R.L., Wilson V.S. (2003). Effects of the androgenic growth promoter 17-β-trenbolone on fecundity and reproductive endocrinology of the fathead minnow. Environ. Toxicol. Chem..

[17] Ankley G.T., Bennett R.S., Erickson R.J., Hoff D.J., Hornung M.W., Johnson R.D., Mount D.R., Nichols J.W., Russom C.L., Schmieder P.K. (2010). Adverse outcome pathways: A conceptual framework to support ecotoxicology research and risk assessment. Environ. Toxicol. Chem..

[18] Burden N., Mahoney C., Müller B.P., Terry C., Westmoreland C., Kimber I. (2015). Aligning the 3Rs with new paradigms in the safety assessment of chemicals. Toxicology.

[19] Tollefsen K.E., Scholz S., Cronin M.T., Edwards S.W., de Knecht J., Crofton K., Garcia-Reyero N., Hartung T., Worth A., Patlewicz G. (2014). Applying Adverse Outcome Pathways (AOPs) to support Integrated Approaches to Testing and Assessment (IATA). Regul. Toxicol. Pharm..

[20] Forbes V.E., Palmqvist A., Bach L. (2006). The use and misuse of biomarkers in ecotoxicology. Environ. Toxicol. Chem..

[21] Taylor N.S., White T.A., Viant M.R. (2017). Defining the Baseline and Oxidant Perturbed Lipidomic Profiles of Daphnia magna. Metabolites.

[22] Ramirez T., Daneshian M., Kamp H., Bois F.Y., Clench M.R., Coen M., Donley B., Fischer S.M., Ekman D.R., Fabian E. (2013). Metabolomics in toxicology and preclinical research. Altex-Altern. Anim. Exp..

[23] Robertson D.G., Watkins P.B., Reily M.D. (2011). Metabolomics in toxicology: Preclinical and clinical applications. Toxicol. Sci..

[24] Viant M.R., Sommer U. (2013). Mass spectrometry based environmental metabolomics: A primer and review. Metabolomics.

[25] Van Ravenzwaay B., Sperber S., Lemke O., Faulhammer F., Kamp H., Mellert W., Strauss V., Strigun A., Peter E., Spitzer M. (2016). Metabolomics as read-across tool: A case study with phenoxy herbicides. Regul. Toxicol. Pharmacol..

[26] Thomas R.S., Clewell H.J., Allen B.C., Wesselkamper S.C., Wang N.C.Y., Lambert J.C., Hess-Wislon J.K., Zhao Q.J., Anderson M.E. (2011). Application of transcriptional benchmark dose values in quantitative cancer and noncancer risk assessment. Toxicol. Sci..

[27] Farmahin R., Williams A., Kuo B., Chepelev N.L., Thomas R.S., Barton-Maclaren T.S., Curran I.H., Nong A., Wade M.G., Yauk C.L. (2017). Recommended approaches in the application of toxicogenomics to derive points of departure for chemical risk assessment. Arch. Toxicol..

[28] Martyniuk C.J., Griffitth R.J., Denslow N.D. (2011). Omics and environmental science. Environ. Toxicol. Chem..

[29] Taylor N.S., Weber R.J.M., White T.A., Viant M.R. (2010). Discriminating between different acute chemical toxicities via changes in the daphnid metabolome. Toxicol. Sci..

[30] De Coen W.M., Janssen C.R. (2003). A multivariate biomarker-based model predicting population-level responses of *Daphnia magna*. Environ. Toxicol. Chem..

[31] Hines A., Staff F.J., Widdows J., Compton R.M., Falciani F., Viant M.R. (2010). Discovery of metabolic signatures for predicting whole organism toxicology. Toxicol. Sci..

[32] Taylor N.S., Kirwan J.A., Johnson C., Yan N.D., Viant M.R., Gunn J.M., McGeer J.C. (2016). Predicting chronic copper and nickel reproductive toxicity to *Daphnia pulex-pulicaria* from whole animal metabolic profiles. Environ. Pollut..

[33] Shaw J.R., Pfrender M.E., Eads B.D., Klaper R., Callaghan A., Sibly R.M., Colson I., Jansen B., Gilbert D., Colbourne J.K., Hogstrand C., Kille P. (2008). Daphnia as an emerging model for toxicological genomics. Comparative Toxicogenomics.

[34] Lankadurai B.P., Nagato E.G., Sinmpson M.J. (2013). Environmental metabolomics: An emerging approach to study organism responses to environmental stressors. Environ. Rev..

[35] Kovacevic V., Simpson A.J., Simpson M.J. (2016). ^1^H NMR-based metabolomics of *Daphnia magna* responses after sub-lethal exposure to triclosan, carbamazepine and ibuprofen. Comp. Biochem. Physiol. Part D Genom. Proteom..

[36] Scanlan L.D., Loguinov A.V., Teng Q., Antczak P., Dailey K.P., Nowinski D.T., Kornbluh J., Lin X.X., Lachenauer E., Arai A. (2015). Gene transcription, metabolite and lipid profiling in eco-indicator *Daphnia magna* indicate diverse mechanisms of toxicity by legacy and emerging flame retardants. Environ. Sci. Technol..

[37] Stanley J.K., Perkins E.J., Habib T., Sims J.G., Chappell P., Escalon B.L., Wilbanks M., Garcia-Reyero N. (2013). The Good, The Bad, and The Toxic: Approaching hormesis in *Daphnia magna* exposed to an energetic compound. Environ. Sci. Technol..

[38] Taylor N.S., Weber R.J.M., Southam A.D., Payne T.G., Hrydziuszko O., Arvanitis T.N., Viant M.R. (2009). A new approach to toxicity testing in *Daphnia magna*: Application of high throughput FT-ICR mass spectrometry metabolomics. Metabolomics.

[39] Sumner L.W., Amberg A., Barrett D., Beale M.H., Beger R., Daykin C.A., Fan T.W.-M., Fiehn O., Goodacre R., Griffin J.L. (2007). Proposed minimum reporting standards for chemical analysis. Metabolomics.

[40] Gillard J., Frenkel J., Devos V., Sabbe K., Paul C., Rempt M., Inzé D., Pohnert G., Vuylsteke M., Vyverman W. (2013). Metabolomics Enables the Structure Elucidation of a Diatom Sex Pheromone. Angew. Chem. Int. Ed. Engl..

[41] Denslow N., Colbourne J.K., Dix D., Freedman J.H., Helbing C.C., Kennedy S., Williams P.L., Di Giulio R., Benson W.H. (2007). Selection of surrogate animal species for comparative toxicogenomics. Genomic Approaches for Cross-Species Extrapolation in Toxicology.

[42] Lampert W. (2011). Daphnia: Development of a Model Organism in Ecology and Evolution.

[43] U.S. Department of Health and Human Services: National Institutes of Health. https://www.nigms.nih.gov/Research/models/Pages/default.aspx.

[44] Garreta-Laura E., Campos B., Barata C., Lacorte S., Tauler R. (2016). Metabolic profiling of *Daphnia magna* exposed to environmental stressors by GC-MS and chemometric tools. Metabolomics.

[45] Southam A.D., Payne T.G., Cooper H.J., Arvanitis T.N., Viant M.R. (2007). Dynamic Range and Mass Accuracy of Wide-Scan Direct Infusion Nanoelectrospray Fourier Transform Ion Cyclotron Resonance Mass Spectrometry-Based Metabolomics Increased by the Spectral Stitching Method. Anal. Chem..

[46] Hao J., Liebeke M., Sommer U., Viant M.R., Bundy J.G., Ebbels T.M.D. (2016). Statistical correlations between NMR spectroscopy and direct infusion FT-ICR mass spectrometry aid annotation of unknowns in metabolomics. Anal. Chem..

[47] Jones M.R., Lawson T.N., Weber R.J.M., Dunn W.B., Viant M.R. (2018). Deep Metabolome Annotation of the Model Organism *Daphnia magna*.

[48] Edison A.S., Hall R.D., Junot C., Karp P.D., Kurland I.J., Mistrik R., Reed L.K., Saito K., Salek R.M., Steinbeck C. (2016). The time is right to focus on model organism metabolomes. Metabolites.

[49] Fontana L., Partridge L. (2015). Promoting health and longevity through diet: From model organisms to humans. Cell.

[50] Smirnoff N., Pallanca J.E. (1996). Ascorbate metabolism in relation to oxidative stress. Biochem. Soc. Trans..

[51] Frei B., England L., Ames B.N. (1989). Ascorbate is an outstanding antioxidant in human blood plasma. Proc. Natl. Acad. Sci. USA.

[52] Ercal N., Gurer-Orhan H., Aykin-Burns N. (2001). Toxic metals and oxidative stress part 1: Mechanisms involved in metal-induced oxidative damage. Curr. Top. Med. Chem..

[53] Lester R., Carey M.C., Little J., Cooperstein L.A., Down S.R. (1975). Crustacean intenstinal detergent promotes sterol solubilisation. Science.

[54] Miyachi S., Miyachi S. (1966). Sulfolipid metabolism in *Chlorella*. Plant Physiol..

[55] Van den Oord A., Danielsson H., Ryhage R. (1965). On the structure of the emulsifiers in gastric juice from the crab, *Cancer pagurus* L.. J. Biol. Chem..

[56] Collatz K.-G., Mommsen G., Mommsen T. (1975). Alkylsulfuric acid and glyceride type emulsifiers in the intestinal juice of the snail *Helix pomatia* L.. J. Comp. Physiol..

[57] De Coen W., Janssen C.R. (2003). The missing biomarker link: Relationships between effects on the cellular energy allocation biomarker of toxicant-stressed *Daphnia magna* and corresponding population characteristics. Environ. Toxicol. Chem..

[58] MEtabolomics standaRds Initiative in Toxicology (MERIT) Project. http://www.ecetoc.org/topics/standardisation-metabolomics-assays-regulatory-toxicology/.

[59] Terry A. (2018). Personal communication.

[60] Weber R.J.M., Viant M.R. (2010). MI-Pack: Increased confidence of metabolite identification in mass spectra by integrating accurate masses and metabolic pathways. Chemometr. Intell. Lab..

[61] Wold S., Sjostrom M., Eriksson L. (2001). PLS-regression: A basic tool of chemometrics. Chemometr. Intell. Lab..

[62] Westerhuis J.A., Hoefsloot H.C.J., Smit S., Vis D.J., Smilde A.K., van Velzen E.J.J., van Duijnhoven J.P.M., van Dorsten F.A. (2008). Assessment of PLSDA cross validation. Metabolomics.

