# Supplementary material for: Metabolomics Discovers Early-Response Metabolic Biomarkers that Can Predict Chronic Reproductive Fitness in Individual Daphnia magna
Source: Metabolites. 2018 Jul 23;8(3):42. doi: 10.3390/metabo8030042 (PMC6160912; doi:10.3390/metabo8030042)
Supplement: Supplementary file 1 [file metabolites-08-00042-s001.zip › Supplemetary information _Metabolites.pdf]

Supplementary Information

Metabolomics discovers *early-response* metabolic biomarkers that can predict *chronic* reproductive fitness in individual *Daphnia magna*

Nadine S. Taylor, Alex Gavin and Mark R. Viant

School of Biosciences, University of Birmingham, Edgbaston, Birmingham B15 2TT, UK

Correspondence: m.viant@bham.ac.uk; Tel.: +44-121-414-2219

Contents of this document:

**Supplemental methods:**<br>Determining of the *D. magna* neonatal LC_{50} concentrations<br>Determining of dose range for chronic (21-day) *D. magna* exposure studies<br>Determining the effects of cadmium, 2,4-dinitrophenol (DNP) and propranolol on the metabolome of *D. magna* following an acute exposure	Page 2
**Table S1**<br>Summary of exposure concentrations for Cd, DNP and propranolol	Page 3
**Supplemental methods:**<br>Perturbation of the *D. magna* metabolome following exposure to cadmium, DNP and propranolol<br>Effect of reduced-food on the *D. magna* metabolome	Page 3
**Table S2**<br>Total number of peaks detected and the proportion of peaks that significantly changed intensity (FDR < 5%) between the control and high dose groups, following chronic exposure of *D. magna* to Cd, DNP and propranolol.	Page 3
**Table S3.**<br>Summary of p-values from t-tests of PC scores between exposed and reduced-food samples, for Cd, DNP and propranolol exposures	Page 4
**Table S4.**<br>Summary of the 49 metabolic features (or peaks) that constitute the *non-*chemical-specific metabolic biomarker signature	Page 5
**Table S6.**<br>Effects of chemical exposure on *Daphnia* glutathione levels.	Page 7

See also supplementary file:

Table S5 Supplementary Information.xlsx
Summary of all annotations for the 49 metabolic features (or peaks) that constitute the *non-*chemical-specific metabolic biomarker signature.

Determining the *D. magna* neonatal LC_{50} concentrations

A study previously published by our laboratory has reported the experimental LC_{50} values for the chemicals used in this study [1]. Exposure conditions were as described, in brief, 24-hour acute toxicity studies were performed for each of the three chemicals (cadmium, 2,4-dinitrophenol (DNP) and propranolol) using groups of 30 neonates (<24 hours old) in 250 mL of clean media with no food or supplements provided during this exposure. Neonatal 24-hour LC_{50} values were determined using PROBIT analysis (SPSS v16, Chicago) and the concentrations were 713.6 µg/L Cd (measured as Cd^{2+} ions), 14.9 mg/L DNP and 13.8 mg/L propranolol.

Determining the dose range for chronic (21-day) *D. magna* exposure studies

The preliminary 21-day exposures of *D. magna* to both DNP and propranolol determined that 10% of the neonatal LC_{50} did not induce any mortality for the duration of the exposure. However, exposure to Cd did induce mortality at 10% of the neonatal LC_{50} concentration, with 100% survival for the duration of a chronic 21-day study only occurring at 1% of the neonatal LC_{50} (7 µg/L). It should be noted that subjective observations of the animals exposed to Cd showed them to be relatively immobile and pale in colour at the end of this period. This preliminary study determined the nominal concentrations to be used in the reported chronic study (Table S1).

Determining the effects of cadmium, 2,4-dinitrophenol (DNP) and propranolol on the metabolome of *D magna* following an acute exposure

A major objective of our previously reported toxicity study [1] was to discover the molecular perturbations to the metabolome of *D. magna* following a short term (24 hours) exposure. While the original study used both whole organism homogenates and haemolymph samples to assess the metabolome, only the data generated from the whole organism homogenate samples are used in the current study as they match the sample type used in the chronic toxicity experiment. The methods used in the original study are detailed in Taylor et al. 2011 [1], and a summary of the experimental conditions is provided below.

Daphniids were cultured as described in the current paper. For the toxicity exposures, third brood neonates (<24 hours old) were cultured until 14 days of age, and then individual daphniids were transferred to 250 mL clean media and exposed to the relevant toxicant for 24 hours, during which time no food or supplements were provided. The experimental design consisted of control animals (n = 10 individuals) and toxicant exposed animals (n = 10 for each of the three toxicants). All toxicants were solubilized in deionized water. The nominal exposure concentration for each of the four toxicants was standardized to 10% of the previously determined neonatal LC_{50} (see above), effectively normalizing the exposure concentration to a biological effect; the concentrations were 71 µg/L Cd (measured as Cd^{2+} ions), 1.5 mg/L DNP and 1.4 mg/L propranolol. Following the 24-hour exposure period, animals were captured and flash frozen in liquid nitrogen, as detailed in the current paper.

Table S1. Summary of exposure concentrations for Cd, DNP and propranolol (n=8 per concentration), as used in the chronic reproductive toxicity assays. Exposure concentrations are also presented in relation to the reported LC_{50} values for each chemical.

Chemical	Nominal concentration	Percentage of neonatal LC_{50} [1]
Cadmium	0.35 µg/L	0.05%
	1.4 µg/L	0.2%
	3.5 µg/L	0.5%
	7.0 µg/L	1%
DNP	0.15 mg/L	1%
	0.75 mg/L	5%
	1.5 mg/L	10%
Propranolol	0.14 mg/L	1%
	0.7 mg/L	5%
	1.4 mg/L	10%

No mortality occurred during the 21-day exposure to propranolol. Some mortality occurred during the chronic exposures in both the Cd and DNP studies. Specifically, during the Cd exposure: 1 animal died in each of the 0 and 1.4 µg/L doses, 5 animals died at 3.5 µg/L, and 3 animals died at 7.0 µg/L. Due to the high levels of mortality in the Cd exposure, the 3.5 and 7.0 µg/L doses were subsequently grouped together as one 'high' dose for the analysis of reproductive output. During the DNP exposure, 1 animal died in each of the 0, 0.15 and 0.75 mg/L dose groups.

Perturbation of the *D. magna* metabolome following exposure to cadmium, DNP and propranolol

Table S2 summarises the total number of peaks observed in each of the three chemical exposure studies, along with the proportion of those peaks that changed intensity significantly in the high dose group relative to untreated controls (Student's t-tests adjusted for FDR < 5%). Consistent with the PCA results presented in the main paper (Figure 2), for which little class separation was visible for DNP, the proportion of significant peaks in the DNP dataset is only 2.6% of the total number of peaks detected. The much higher proportion of significant peaks in both the Cd and propranolol datasets (59% and 44% respectively) is again consistent with the much more evident class separation seen in the corresponding PCA scores plots (Figure 2).

Table S2. Total number of peaks detected and the proportion of peaks that significantly changed intensity (FDR < 5%) between the control and high dose groups, following chronic exposure of *D. magna* to Cd, DNP and propranolol.

	Cd	DNP	Propranolol
**Total number of peaks observed**	4056	4112	3647
**Proportion of significant peaks (%)**	59	2.6	44

It should be noted that the putative annotation of these peaks following data processing allowed for the identification of two peaks corresponding to DNP, specifically the $[DNP\text{-}H]^-$ and $[DNP(^{13}C)\text{-}H]^-$ ion forms. Since these peaks arise from the parent chemical, they were removed from the dataset prior to any statistical analysis. Neither Cd nor propranolol was detected.

Effect of reduced-food on the *D. magna* metabolome

Statistical analysis of the PC scores data (from the PCAs of the metabolomics data, Figure 2) revealed that the reduced-food group was significantly different from the low, medium and high dose exposure groups, for each of the three chemicals in the study (t-tests with Bonferroni-corrected *p*-value < 0.0125).

Table S3. Summary of *p*-values from t-tests of PC scores between exposed and reduced-food samples, for Cd, DNP and propranolol exposures (data from Figure 2).

Chemical	Reduced food vs. dose group	*p*-value	PC scores
Cadmium	High dose	1.5×10^{-13}	PC1
	Low/Medium dose	3.3×10^{-4}	PC2
DNP	Low/Medium/High	6.0×10^{-7}	PC1
Propranolol	Medium/High	1.1×10^{-15}	PC1
	Low	3.1×10^{-12}	PC2

Table S4. Summary of the 49 metabolic features (or peaks) that constitute the *non*-chemical-specific metabolic biomarker signature. The peaks are ranked in order of importance according to the forward selected PLS-R model. Also included in the table are the *m/z* values, average intensities and the fold-change in intensities between the high dose and control groups for each of the three chemical exposure datasets.

Rank order of peak importance in PLS-R model	Cd			DNP			Propranolol		
	Measured m/z	Average intensity	Fold change (high dose / control)	Measured m/z	Average intensity	Fold change (high dose / control)	Measured m/z	Average intensity	Fold change (high dose / control)
1	441.17272	3.94E+04	0.534	441.17270	8.26E+04	0.722	441.17276	5.49E+04	1.011
2	228.04585	3.49E+04	1.670	228.04763	1.98E+05	1.554	228.04755	2.56E+05	0.074
3	215.03828	2.72E+05	2.456	215.03834	8.23E+04	1.776	215.03830	8.76E+04	0.489
4	326.11080	2.42E+05	5.059	326.11079	1.09E+05	1.474	326.11073	2.97E+05	5.984
5	175.02481	2.86E+04	0.498	175.02480	3.15E+04	0.636	175.02482	2.86E+04	0.571
6	258.10637	2.97E+04	4.092	258.10642	1.57E+05	0.518	258.10635	3.12E+04	1.049
7	441.01832	6.80E+04	26.168	441.01813	2.05E+04	0.987	441.01816	4.02E+04	5.705
8	274.03900	3.01E+04	1.062	274.03899	5.46E+04	0.370	274.03899	7.39E+04	0.519
9	263.13221	5.00E+05	1.853	263.13227	3.32E+05	2.099	263.13221	5.06E+05	2.898
10	478.98683	1.28E+05	21.172	478.98670	3.49E+04	0.780	478.98663	9.63E+04	5.874
11	310.11436	6.95E+04	0.527	310.11427	2.10E+05	0.309	310.11436	5.05E+05	0.407
12	396.98317	2.08E+05	16.046	396.98290	6.76E+04	0.907	396.98310	1.49E+05	4.722
13	257.10302	2.24E+05	4.155	257.10306	9.00E+05	0.537	257.10301	1.88E+05	0.917
14	309.03348	5.77E+04	0.248	309.03341	1.31E+05	1.089	309.03350	6.06E+04	0.450
15	401.99766	1.74E+04	0.468	401.99750	1.74E+04	1.433	401.99751	1.09E+04	0.404
16	237.99438	1.32E+05	1.354	237.99446	2.85E+05	0.386	237.99440	1.60E+05	7.225
17	295.12201	2.86E+04	0.428	295.12191	3.31E+04	0.606	295.12202	5.66E+04	0.307
18	310.96619	5.60E+04	0.187	310.96615	7.81E+04	0.976	310.96630	5.56E+04	0.382
19	310.11747	2.77E+04	0.614	310.11735	8.04E+04	0.236	310.11741	2.29E+05	0.237
20	392.96926	4.06E+04	0.128	392.96904	4.23E+04	1.045	392.96899	2.89E+04	0.204
21	355.12463	2.19E+04	2.308	355.12469	3.87E+04	3.250	355.12465	5.15E+04	9.007
22	474.97252	2.43E+04	0.182	474.97227	2.82E+04	1.030	474.97212	1.86E+04	0.209
23	242.01531	3.24E+04	0.607	242.01539	1.02E+05	2.014	242.01532	6.55E+04	1.145

24	331.05933	1.85E+04	0.141	331.05934	1.83E+04	0.313	331.05929	1.11E+04	0.342
25	252.13555	5.42E+05	0.355	252.13562	6.72E+05	0.635	252.13557	2.40E+05	0.373
26	251.09578	3.11E+05	0.503	251.09579	2.98E+05	1.163	251.09574	5.96E+05	0.188
27	295.99254	1.64E+04	0.438	295.99254	1.85E+04	0.974	295.99264	1.46E+04	0.383
28	425.13040	3.42E+04	1.722	425.13033	6.69E+04	3.164	425.13032	7.77E+04	8.740
29	287.01825	8.77E+03	2.114	287.01810	3.35E+04	1.531	287.01813	2.72E+04	0.877
30	372.10751	3.17E+04	0.186	372.10738	5.94E+04	0.832	372.10739	2.63E+04	0.211
31	243.00905	1.20E+05	0.616	243.00911	3.80E+05	2.266	243.00905	2.51E+05	1.313
32	458.99866	3.22E+04	0.577	458.99847	4.38E+04	0.938	458.99847	3.86E+04	0.418
33	454.94205	2.98E+04	17.413	454.94193	1.22E+04	0.782	454.94190	3.01E+04	4.776
34	400.14608	2.18E+04	20.737	400.14602	1.65E+04	2.146	400.14625	4.16E+04	7.558
35	247.06182	4.26E+04	3.585	247.06191	1.57E+05	1.982	247.06185	2.06E+05	1.681
36	457.20404	3.67E+04	1.172	457.20380	6.42E+04	3.858	457.20396	1.34E+05	9.648
37	372.11548	1.78E+04	0.454	372.11494	1.13E+04	0.689	372.11549	4.24E+04	0.220
38	359.16755	8.34E+03	3.428	359.16749	1.95E+04	0.999	359.16752	1.03E+04	0.545
39	391.01843	9.97E+03	0.616	391.01877	1.19E+04	0.638	391.01834	1.33E+04	0.710
40	443.14090	4.50E+04	1.863	443.14059	7.89E+04	2.445	443.14080	1.11E+05	6.254
41	294.99218	1.62E+05	0.214	294.99208	1.78E+05	0.956	294.99217	1.36E+05	0.424
42	282.95769	4.03E+04	0.754	282.95765	3.03E+04	0.538	282.95758	2.97E+04	0.739
43	258.05639	7.84E+04	0.931	258.05642	1.11E+05	0.307	258.05634	2.71E+05	0.668
44	340.12478	1.68E+04	11.993	340.12479	1.77E+04	2.248	340.12493	2.62E+04	5.284
45	464.97106	1.79E+04	12.919	464.97088	9.90E+03	0.792	464.97086	1.73E+04	3.425
46	404.04780	4.33E+04	0.779	404.04758	4.37E+04	1.121	404.04770	3.20E+04	0.863
47	352.04448	3.09E+04	0.670	352.04440	2.76E+04	1.473	352.04435	1.33E+04	0.890
48	421.00589	3.81E+04	1.084	421.00562	3.36E+04	1.362	421.00562	2.76E+04	1.519
49	415.14615	5.41E+04	1.193	415.14588	8.35E+04	1.737	415.14590	1.41E+05	2.582

Table S6. Effects of chemical exposure on *Daphnia* glutathione levels.

	Ion form	Measured *m/z*	*m/z* error (ppm)	Fold change (high dose / control)
Cd	[M-H]-	306.0765	-0.11	0.603
	[M+Na-2H]-	328.05856	0.25	0.649
	[M+Cl]-	342.05321	0.00	0.703
				Average = 0.652
DNP	[M-H]-	306.07641	-0.4	1.512
	[M+Na-2H]-	328.05849	0.04	1.592
	[M+Cl]-	342.0532	-0.03	1.543
				Average = 1.549
Propranolol	[M-H]-	306.07652	-0.04	0.865
	[M+Na-2H]-	328.05851	0.1	1.073
	[M+Cl]-	Not observed	-	-
				Average = 0.969

References

[1] Taylor, N.S.; Weber, R.J.M.; White, T.A.; Viant, M.R. Discriminating between different acute chemical toxicities via changes in the daphnid metabolome. *Toxicol. Sci*. **2010**, *118*, 307-317.

[2] Southam, A.D.; Payne, T.G.; Cooper, H.J.; Arvanitis, T.N.; Viant, M.R. Dynamic Range and Mass Accuracy of Wide-Scan Direct Infusion Nanoelectrospray Fourier Transform Ion Cyclotron Resonance Mass Spectrometry-Based Metabolomics Increased by the Spectral Stitching Method. *Anal. Chem*. **2007**, *79*, 4595-4602.
